# The Fraser Complex Proteins (Frem1, Frem2, and Fras1) Can Form Anchoring Cords in the Absence of AMACO at the Dermal–Epidermal Junction of Mouse Skin

**DOI:** 10.3390/ijms24076782

**Published:** 2023-04-05

**Authors:** Temitope Esho, Birgit Kobbe, Sara F. Tufa, Douglas R. Keene, Mats Paulsson, Raimund Wagener

**Affiliations:** 1 Center for Biochemistry, Medical Faculty, University of Cologne, 50931 Cologne, Germany; 2 Micro-Imaging Center, Shriners Children’s, Portland, OR 97239, USA; 3 Center for Molecular Medicine Cologne, 50931 Cologne, Germany; 4 Cologne Center for Musculoskeletal Biomechanics, 50931 Cologne, Germany

**Keywords:** anchoring cords, AMACO, Fraser complex proteins, extracellular matrix, basement membrane, immunogold electron microscopy

## Abstract

AMACO (VWA2 protein), secreted by epithelial cells, is strongly expressed at basement membranes when budding or invagination occurs in embryos. In skin, AMACO associates with proteins of the Fraser complex, which form anchoring cords. These, during development, temporally stabilize the dermal–epidermal junction, pending the formation of collagen VII-containing anchoring fibrils. Fraser syndrome in humans results if any of the core members of the Fraser complex (Fras1, Frem1, Frem2) are mutated. Fraser syndrome is characterized by subepidermal blistering, cryptophthalmos, and syndactyly. In an attempt to determine AMACO function, we generated and characterized AMACO-deficient mice. In contrast to Fraser complex mutant mice, AMACO-deficient animals lack an obvious phenotype. The mutually interdependent basement membrane deposition of the Fraser complex proteins, and the formation of anchoring cords, are not affected. Furthermore, hair follicle development in newborn AMACO-deficient mice showed no gross aberration. Surprisingly, it appears that, while AMACO is a component of the anchoring cords, it is not essential for their formation or function.

## 1. Introduction

AMACO (containing A domains related to those in matrilins and collagens; also VWA2 protein) is a basement membrane associated protein, that is secreted by epithelial cells in many organs. In the embryo, it is strongly expressed in heart, condensing somites, choroid plexus, cochlea, terminal bronchii of the lung, skin, developing teeth, and in the oral cavity [1]. It is strongly expressed when invagination or budding occurs during development, was found as a molecular signature of hair placodes [2], and is strikingly upregulated in the mid-telogen phase of the hair cycle [3]. During development in zebrafish and mice, AMACO (encoded by the VWA2 gene) co-localizes at the basement membrane with the Fraser complex proteins. Strikingly, AMACO deposition is absent in Fras1-deficient zebrafish and mice. Additionally, the morpholino knockdown of AMACO strengthens the phenotype of hypomorphic Fras1 mutant zebrafish [4]. This indicates that AMACO plays a role in the formation of the Fraser complex, and AMACO has been designated the newest member of this complex [5]. However, no mutation in AMACO has been associated with the Fraser syndrome or blebs mutant phenotypes.

The human VWA2 gene is 51 kb, while the mouse gene is 37 kb long. They both comprise 13 exons, that code for the translated sections of the mRNA. As expected, the genes are located on syntenic regions of chromosome 10 (10q26.11) and 19 (19D2), respectively, and have identical exon–intron organizations [1]. AMACO contains an N-terminal VWA domain (VWA1), followed by a cysteine-rich domain, that does not have any apparent similarity to other known protein domains. Towards the C-terminus, it follows a non-calcium-binding epidermal growth factor like domain (EGF1), carrying a characteristic O-fucosylation and O-glucosylation [6], and an additional tandem pair of VWA domains (VWA2 and VWA3). A second non-calcium-binding EGF domain (EGF2), is located close to the C-terminus, that is a unique region in mice [1]. In humans, the unique region is only partially present, due to a frameshift that follows a 5 bp gene deletion, resulting in a premature stop codon [1]. The final protein has a calculated molecular weight of 79,485 in humans and 83,024 in mice. The domain structure of AMACO is entirely conserved between mammals, birds, amphibians, and zebrafish [7], and human and mouse AMACO are 80% identical at the protein level [1]. There is no other AMACO-like gene present in the fully sequenced human and mouse genomes, making AMACO the only member of a new subfamily, belonging to the minority of VWA family members that do not have a paralog [1]. In humans, AMACO was also referred to as colon cancer secreted protein-2 (CCSP-2), as it was found to be upregulated in colon cancer [8], and epigenetic and transcriptional dysregulation of VWA2 was found to be associated with an MYC-driven oncogenic program in colorectal cancer [9].

AMACO is part of the Fraser complex, a group of proteins which, when mutated in humans, cause the Fraser syndrome, with phenotypes such as cryptophthalmos, syndactyly, and other developmental impairments, as a consequence of embryonic subepithelial blister formation [10]. By immunofluorescence microscopy, the core Fraser complex proteins Fras1, Frem1 (Fras1 related extracellular matrix protein 1), and Frem2, all localize beneath the lamina densa, where they mediate adhesion at the dermal–epidermal junction (DEJ) during embryonic development [11]. Fras1 is the prototypic Fraser complex protein and has been well characterized. It has about 4000 amino acid residues and a multidomain structure, consisting of five von Willebrand factor C-like (VWC) at the N-terminus, followed by five furin-like, 15 CSPG-like, five CALX-β domains, and a unique, a transmembrane, and a cytosolic domain at the C-terminus. It is expressed in adult kidney, pancreas, and thalamus, and highly expressed in fetal kidney and heart. A particularly high expression was observed in the apical ectodermal ridge of the limb buds at E10.5–E12.5 and the interdigital spaces at E14.5. It is present underlying the surface epithelium of the entire embryo, and in the linings of the peritoneal cavity and dorsal aorta [12]. Frem1, Frem2, and Frem3 share the 15 CSPG-like domains and a variable number of CALX-β domains, but lack the VWC and furin-like domains; also, Frem1 does not have a transmembrane domain [11]. In contrast to Frem1 and AMACO, both Fras1 and Frem2 are first expressed as transmembrane proteins, that are later shed from the plasma membrane [13,14,15]. Frem3 localizes independently of the Fras1/Frem1/Frem2 protein complex, within the sublamina densa [16]. Ablation of Frem3 in mice does not lead to Fraser syndrome [17]. Nephronectin is also associated with core Fraser complex proteins and has a similar expression at basement membranes of developing organs, such as eyes, lungs, teeth, hair, taste buds, and kidneys [18]. Indeed, loss of Frem1 significantly diminished the expression of nephronectin [19] and loss of nephronectin leads to phenotypes overlapping with Fraser syndrome phenotypes [20].

Initial studies, using immunogold electron microscopy, of adult mouse skin, showed limited, clustered AMACO deposition below the lamina densa [7]. More recently, the presence of an extended network of cord-like suprastructures in the dermis of newborns was described, to which AMACO, members of the Fraser complex, and nephronectin contribute [21]. To unequivocally designate this novel suprastructure, the name “anchoring cords” was proposed. The AMACO-containing anchoring cords, have a diameter of about 60 nm, originate from the basement membrane, and occasionally extend several microns into the dermis. Following traditional staining with osmium, uranium, and lead, anchoring chords are translucent, and difficult to detect in the transmission electron microscope. They are easily confused with other structures common at the DEJ, including collagen VII-containing anchoring fibrils and fibrillin micofibrils. They are most easily recognized in a mouse model of dystrophic epidermolysis bullosa. In this model, the neonate mouse skin lacks collagen VII, whereby anchoring fibrils are eliminated from the field [21]. However, they become clearly evident with the use of gold-labeled antibodies, in 300 nm thick sections examined in the TEM, whereby gold particles coat the low-density core of the anchoring cords. The Fraser complex members localize preferentially along the length of anchoring cords. Frem1 distribution is limited to the lamina lucida and lamina densa, Fras1 localizes to the region of cords immediately adjacent to the lamina densa, and Frem2 co-localizes with AMACO along the full length of the anchoring cords [21].

Here, AMACO-deficient mice were generated, to provide insight into its function. In contrast to Fraser complex-deficient mice, which display several developmental defects [11], AMACO-deficient mice lack an obvious phenotype. The mutually dependent basement membrane deposition of the Fraser complex proteins, was not perturbed in the absence of AMACO, and unlabeled anchoring cords could be detected by TEM at the dermal–epidermal junction (DEJ). Additionally, investigation of the hair follicle development in newborn AMACO-deficient mice also showed no gross aberration.

## 2. Results

### 2.1. Generation of an AMACO-Deficient Mouse Line

**Figure 1:**
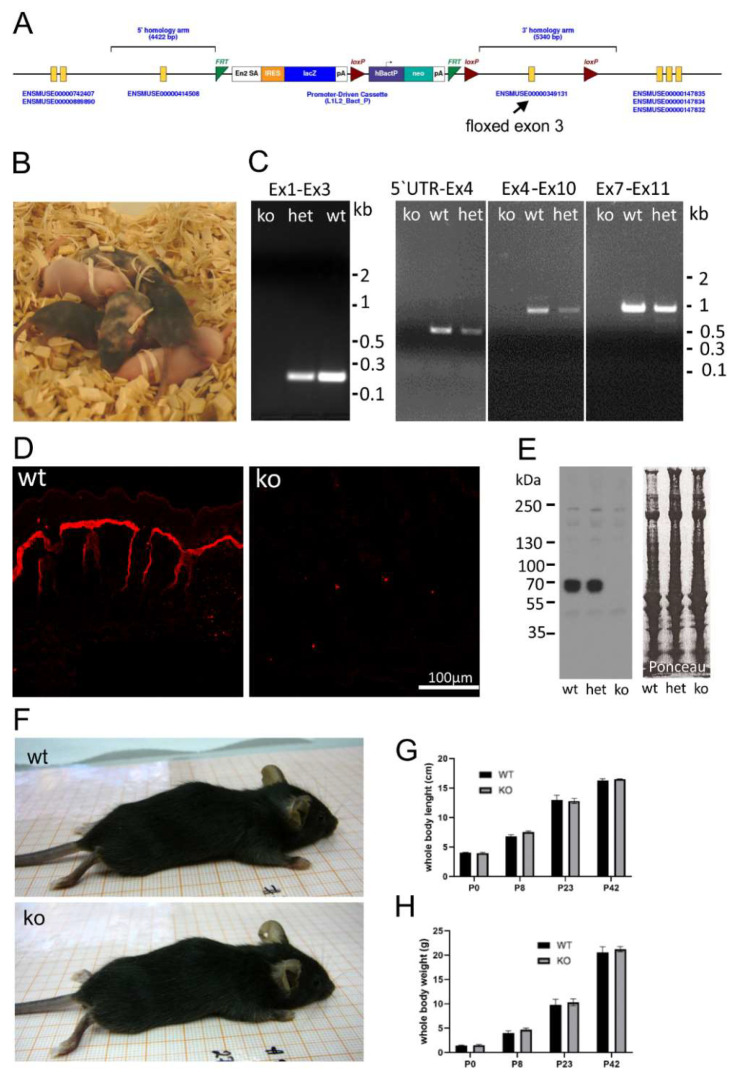
AMACO-deficient mouse model. (**A**) The targeting construct transfected into embryonic stem cells, contained exon 3 flanked by two loxP sites. En2 SA—engrailed 2 splicing acceptor; IRES—internal ribosome entry site; lacZ—β-galactosidase gene; pA—poly A; hbactP—human beta actin promoter; neo—promoter-driven neomycin resistance gene; FRT—flippase recognition target; Loxp—locus of crossover in p1. Modified from the KOMP consortium. (**B**) Five chimeric mice, obtained after successful injection of ES cells into a foster mouse. (**C**) RT-PCR on mRNA, showing the lack of transcribed message of the AMACO gene after successful deletion of the exon 3 gene with a Cre deleter mouse. Primers were designed to amplify different mRNA fragments because amplification of the full fragment was not successful. Ex1–Ex3, 5′UTR-Ex4, Ex4–Ex10, and Ex7–Ex11, correspond to bands of 190 bp, 530 bp, 1040 bp, and 1090 bp, respectively (Ex—exon; het—heterozygous). (**D**) Immunofluorescence analysis of newborn mouse skin showed a complete lack of AMACO in knockout mice. (**E**) Further characterization of the AMACO-knockout skin by immunoblot analysis showed a complete lack of AMACO expression. The blot is overexposed, to unequivocally show the absence of AMACO in knockout animals. Therefore, a possible difference between WT and het cannot be seen. Ponceau staining showed equal loading. (**F**) Physical observation of knockout mice compared to wildtype controls showed no obvious phenotype. Whole body weight (**G**) and length (**H**) recording from birth until adulthood revealed no significant alteration compared to the wildtype controls. *n* = 5 (P—postnatal day).

To investigate the function of AMACO, a null mouse strain was generated. The knockout model was obtained by injection of embryonic stem cells carrying a floxed exon 3 of the AMACO gene (Figure 1A) from the Knockout Mouse Project (KOMP) Repository, into foster mothers, on a C57BL6/J background. Highly chimeric mice were obtained (Figure 1B) and germline transmission established. By crossing with a Cre deleter strain, exon 3 of the AMACO (VWA2) gene was deleted and RT-PCR demonstrated the complete absence of AMACO transcription in the knockout mice (Figure 1C), indicating nonsense-mediated mRNA decay. Genomic PCR fragments, obtained from genotyping primers, were sequenced, and revealed the complete loss of exon 3 and the neo cassette gene (not shown). Immunofluorescence analysis was used to demonstrate the complete loss of AMACO expression in the skin of newborn mice (Figure 1D). This was also shown by immunoblot analysis on newborn skin tissue extracts (Figure 1E).

### 2.2. AMACO Expressing Tissues Display No Obvious Aberrations upon AMACO Deficiency

**Figure 2:**
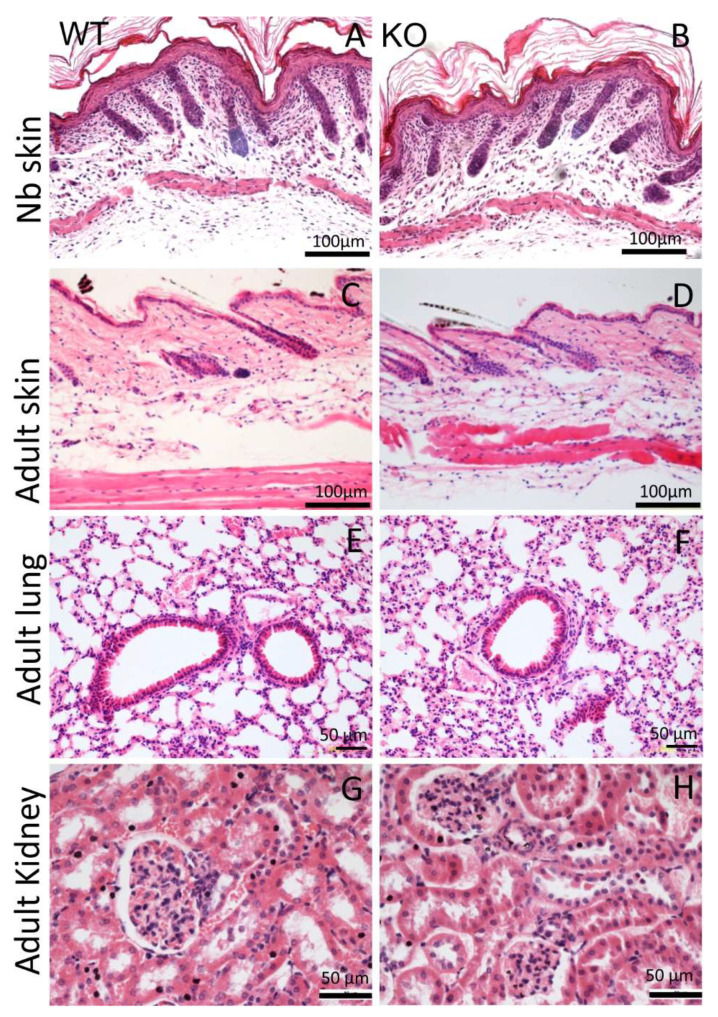
Histological analysis of AMACO-expressing tissues. Hematoxylin/eosin staining of paraffin-embedded sections displayed normal tissue architecture in the AMACO-deficient newborn (**B**) and adult (**D**) skin, and adult lung (**F**) and kidney (**H**). Wildtype controls (**A**,**C**,**E**,**G**). Scale bars = 100 µm in (**A**–**D**) and 50 µm in (**E**–**H**).

The AMACO-deficient mice displayed no overt phenotype upon physical investigation, and were viable and fertile (Figure 1F). Analysis of whole-body length (Figure 1G) and weight (Figure 1H) of the AMACO-deficient mice from birth until adulthood, revealed no significant alteration in comparison to the wildtype control animals. AMACO is expressed in skin, developing teeth, kidneys, and choroid plexus, often when invagination or budding occurs during development [1]. AMACO is strongly expressed on the mesenchymal side, just below the lamina densa of basement membranes. Therefore, sections of embryonal, newborn, and adult AMACO-deficient mice were analyzed by hematoxylin/eosin staining, to investigate tissue morphology and architecture of tissues normally expressing AMACO (Figure 2). Both newborn (Figure 2A,B) and adult (Figure 2C,D) skin displayed normal tissue architecture. Lungs (Figure 2E,F) and kidneys (Figure 2G,H) did not show any observable difference compared to wildtype controls. Other developing tissues, such as intestine, esophagus, and teeth, also did not show any differences from wildtype (not shown). In clear contrast to Fraser complex protein-deficient mice, which show a wide range of developmental defects [11], the AMACO-deficient mice displayed no overt phenotype.

### 2.3. Expression and Basement Membrane Deposition of Fraser Complex Proteins Are Not Affected in AMACO-Deficient Mice

**Figure 3:**
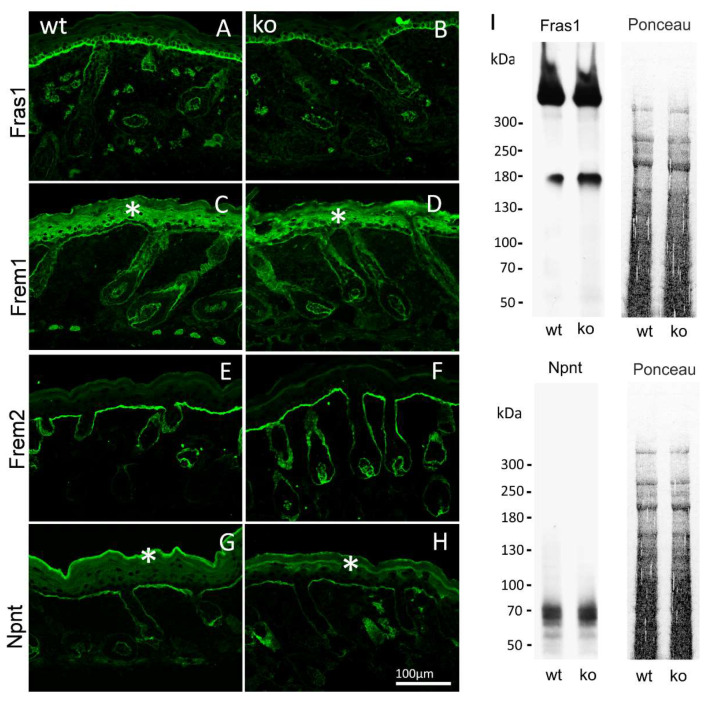
Immunofluorescence and immunoblot analysis of the Fraser complex proteins in AMACO-deficient newborn mouse. Frozen sections of wildtype (**A**,**C**,**E**,**G**) and AMACO-deficient newborn mouse skin (**B**,**D**,**F**,**H**) were incubated with affinity purified Fras1, Frem1, Frem2, and nephronectin antibodies, followed by AlexaFluor 488-labeled secondary antibodies. Asterisks (*) indicate a region of unspecific staining by the Frem1 and nephronectin antibodies. Scale bar = 100 µm for (**A**–**H**). Total head extracts of AMACO-deficient mice analyzed by immunoblot showed comparable signals for Fras1 and nephronectin, in comparison to the wildtype control (**I**). Ponceau staining showed equal loading.

**Figure 4:**
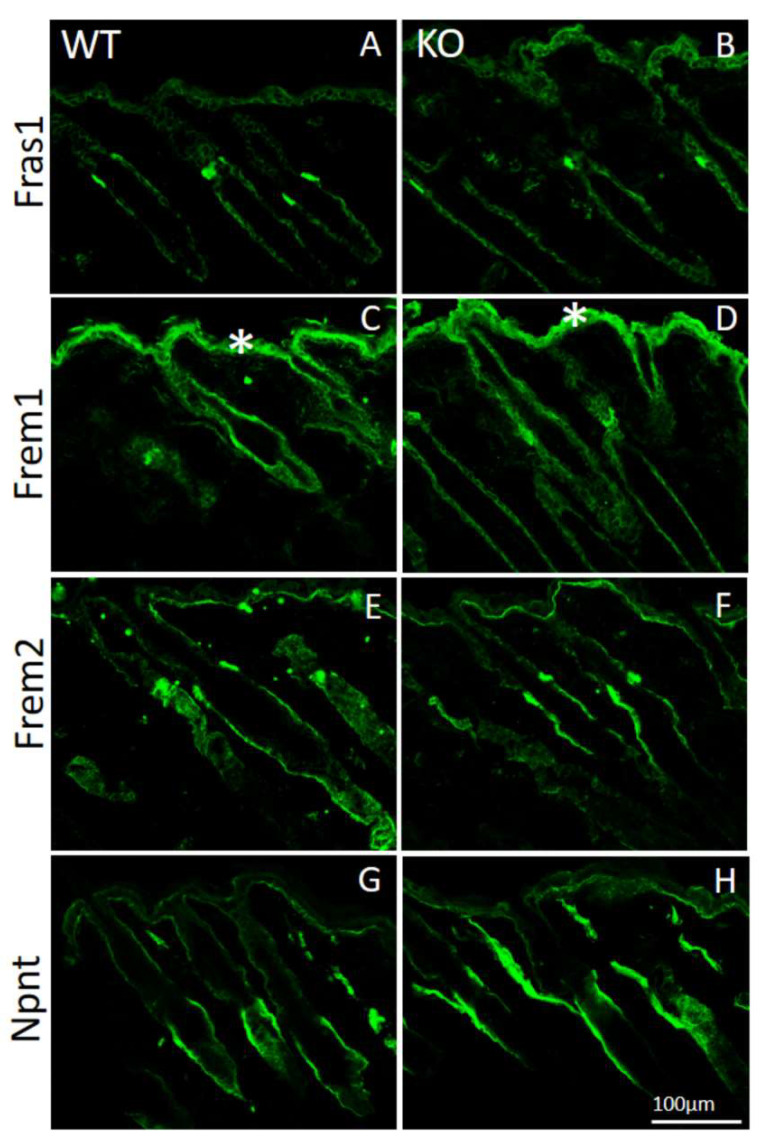
Immunofluorescence analysis of Fraser complex proteins in AMACO-deficient P16 mouse skin. Frozen sections of skin from wildtype (**A**,**C**,**E**,**G**) and AMACO-deficient P16 mice (**B**,**D**,**F**,**H**) were incubated with affinity-purified Fras1, Frem1, Frem2, and nephronectin antibodies, followed by AlexaFluor 488-labeled secondary antibodies. Each Fraser complex component localizes similarly in WT and AMACO-deficient skin. Asterisks (*) show regions of unspecific staining with the Frem1 antibody. Scale bar = 100 µm for (**A**–**H**).

By immunofluorescence microscopy of normal mouse skin, it was seen that the core Fraser complex proteins localize in a similar pattern at epithelial basement membranes [11]. The lack of any one of the components in mice, destabilizes formation of the complex, and therefore results in diverse phenotypes, such as cryptophthalmos, syndactyly, renal agenesis, ambiguous genitalia, and respiratory tract defects [13]. The basement membrane deposition of the Fraser complex proteins in AMACO-deficient mouse skin, was therefore investigated by immunofluorescence analysis. Surprisingly, Fraser complex protein deposition (Fras1, Frem1, Frem2, and nephronectin) was not altered at the basement membrane of newborn (Figure 3) and adult (Figure 4) AMACO-deficient mouse skin, when compared with wildtype. This result is in stark contrast to Fras1-deficient mice, where AMACO and the other Fraser complex proteins are completely or partially translocated from the basement membrane [4,13].

To further investigate the expression and stability of the Fraser complex proteins, extracts of heads of newborn AMACO-deficient mice were evaluated by immunoblot analysis. Comparable signals for Fras1 and nephronectin were obtained in AMACO-deficient newborn heads and wildtype controls (Figure 3I). Taken together, expression and basement membrane deposition of the Fraser complex proteins does not appear to be altered in AMACO-deficient mice, suggesting that AMACO may be a non-essential part of the Fraser complex.

### 2.4. Ultrastructural Localization of Fraser Complex Proteins and Basement Membrane Structure Is Not Altered in AMACO-Deficient Mice

**Figure 5:**
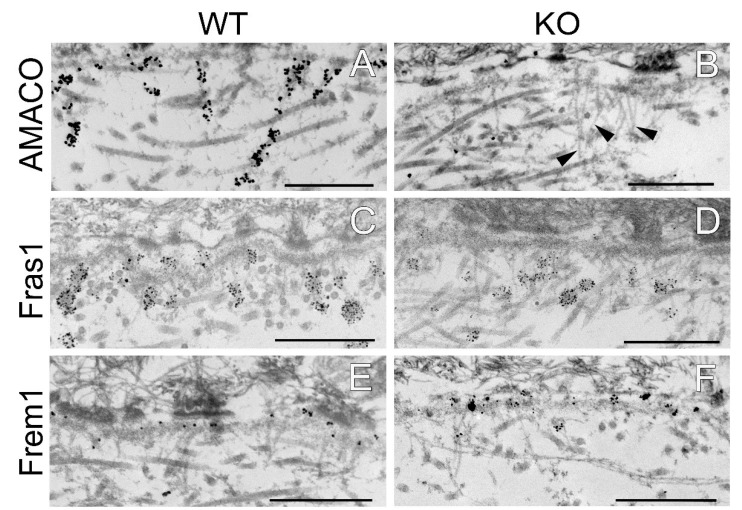

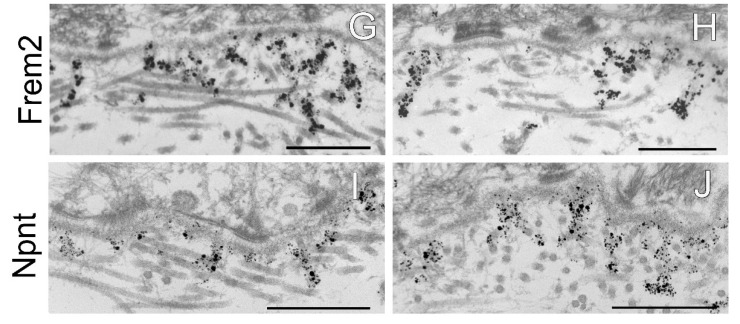
Immunogold electron microscopy analysis of the dermal–epidermal junction in AMACO-deficient newborn mouse skin. (**A**) An AMACO guinea pig antibody directs immunogold to anchoring cords, which insert into the subepithelial basement membrane and extend into the dermis. (**B**) Labeling for AMACO is absent in AMACO-deficient mice skin, however anchoring cords are clearly present (arrows). (**C**,**E**,**G**,**I**) Fras1, Frem1, Frem2, and nephronectin rabbit antibodies detect anchoring cords with a similar distribution as in AMACO-deficient skin (**D**,**F**,**H**,**J**). Scale bars = 500 nm.

The effect of AMACO deficiency on the localization of the Fraser complex members along the length of anchoring cords, was evaluated in newborn mouse skin by immuno-electron microscopy. As expected, AMACO antibody directed gold labeling to anchoring cords in wildtype skin (Figure 5A), and was negative in AMACO-deficient mice (Figure 5B). Importantly, unlabeled anchoring cords could be detected in some regions of the unlabeled AMACO-deficient skin (Figure 5B, arrows). In agreement with histological analysis at the light microscopy level, the basement membrane architecture was not perturbed (Figure 5). Antibodies specific for Fras1, Frem1, Frem2, and nephronectin directed immunogold to their normally expected, relative positions along anchoring cords, directly comparable to the immunolabel seen in wildtype littermates (Figure 5). In conclusion, the basement membrane deposition of the Fraser complex proteins is unaffected by the absence of AMACO, with the stability and architecture of the Fraser complex being independent of AMACO. There is no phenotype detectable resulting from AMACO deficiency.

## 3. Discussion

### 3.1. Genetic Studies of AMACO and Fraser Complex Protein Function

The role of AMACO during development was initially studied in zebrafish, by use of translation inhibiting morpholinos, which sufficiently decreased AMACO protein expression. Morphants displayed normal morphology at 48 and 80 hpf, even in body regions that usually have high AMACO expression levels, such as the pronephros, the fins, the somitic myosepta, and the craniofacial cartilage [4]. These tissues are partly affected in Fras1 mutant zebrafish [22,23]. Surprisingly, Fras1 levels appeared largely normal in AMACO-deficient morphants, in contrast to the loss of AMACO in Fras1-deficient zebrafish [4]. Mutual stabilization of Fras1, Frem1, Frem2, and possibly Frem3 in zebrafish, has been described [22]. The results obtained with the AMACO morphants, suggest that AMACO, *per se*, is dispensable for early zebrafish development. However, AMACO deficiency increases the phenotypic strength in hypomorphic Fras1 zebrafish, so that the fish show a similar phenotype as the complete Fras1 knockout. This suggests that AMACO can stabilize Fras1 at the same time as Fras1 is required for AMACO deposition or stabilization [4]. Overall, the function of AMACO within the Fraser complex remains ambiguous. It was therefore our hope that our AMACO-deficient animal model could help to unravel this function.

### 3.2. Generation and Characterization of an AMACO-Deficient Mouse Line

Mice with a deficiency in the Fraser complex proteins Fras1, Frem1, and Frem2 often die before birth. The few that reach adulthood display phenotypes such as cryptophthalmos, syndactyly, ambiguous genitalia, renal agenesis, and respiratory defects. These phenotypes are accompanied by subepidermal blisters, occurring just below the subepithelial basement membrane [12,13], where type collagen VII and anchoring fibrils form later in development. The blisters present in Fraser complex-deficient mice are similar to the blisters present in dystrophic epidermolysis bullosa, caused by the lack of collagen VII-containing anchoring fibrils [24,25,26]. We postulated that potential blister formation caused by AMACO deficiency, would most likely result from defects in anchoring cords, which may have a similar role as anchoring fibrils, albeit only during fetal development.

Still, AMACO-deficient mice lack any obvious phenotype in AMACO-expressing tissues such as skin, intestine, lungs, esophagus, molars, and kidneys in fetal, newborn, and adult mice. These findings are in contrast to those of Fraser complex-deficient mice, which show several developmental defects [11,13].

The concerted formation of the Fraser complex, explains why the lack of individual members yields comparable phenotypes. Frem3 remains attached to the basement membrane underlying the detached epidermis at the roof of Fras1^−/−^ blistered embryo skin, and is therefore anchored in the extracellular environment independent of Fras1 [16]. This is supported by the fact that Frem3-deficient mice do not develop signs of Fraser syndrome [17], and a human disease due to FREM3 deficiency is not known. Ultrastructural localization of the Fraser complex proteins, and the overall character of the basement membrane zone, is unaffected in AMACO-deficient mouse skin, with the basement membrane architecture remaining comparable to wildtype. Analysis of tissue extracts by immunoblot, showed that the expression and tissue anchorage of the Fraser complex proteins is unaffected in AMACO-deficient mice (Figure 3I).

The absence of AMACO, has little or no effect on the formation and anchorage of the Fraser complex. This challenges our initial hypothesis, that the similarity between AMACO and Frem2 deposition indicates an integral role for AMACO in the formation of anchoring cords. Interestingly, earlier work shows that AMACO tissue distribution is not affected in Frem2a mutant zebrafish. AMACO may be only partially required for the formation and stability of the Fraser complex, so that its integrity is still maintained in the absence of AMACO. The results also suggest that the interaction between AMACO and Frem2 is at the periphery of the complex and that AMACO is connected to the complex via Frem2. Indeed, AMACO antibody directs immunogold labeling at the periphery of the anchoring cords, suggesting that AMACO is distributed only to the surface of anchoring cords [21]. Although AMACO is the only member of the Fraser complex that localizes to the full length of anchoring cords, if AMACO merely coats the outside of the fibrils, perhaps this helps to explain why the lack of AMACO has no significant effect on the Fraser complex.

It is surprising that a unique and well conserved protein gives no obvious phenotype when deficient. The lack of observable defects in AMACO-deficient mice could also indicate a mechanism to compensate for the lack of AMACO, perhaps by other members of the Fraser complex. Another protein that could compensate for AMACO is collagen VII, which forms anchoring fibrils, a component of the DEJ with an important role in maintaining the adhesion of the epithelium with the dermis [27]. Anchoring fibrils first appear in fetal development [28], but defects resulting from collagen VII deficiency occur only after birth. In contrast, defects in Fras1/Frem are present already in utero, from day E12 onwards. This suggests a complementary but temporal difference in function between the two complexes.

### 3.3. AMACO Function under Challenging Conditions and in Disease

The normal development and tissue architecture of AMACO-deficient mice does not rule out that AMACO may function importantly under challenging conditions, such as wound healing or mechanical stress. Immunofluorescence staining on paraffin-embedded sections of small skin wounds in wildtype mice showed no effect on the expression of AMACO within and surrounding the wound areas (not shown). However, in large wounds, such as tail amputation in lizards, AMACO is strongly upregulated during healing and regeneration [29]. Interestingly, anchoring cords were readily found at the DEJ of skin from a mouse model of dystrophic epidermolysis bullosa, lacking anchoring fibrils [21]. That anchoring cords could be found so easily could be explained by the lack of anchoring fibrils, whose structure and position competes with anchoring cords; however, if there was indeed an increase in the number of anchoring cords, this may be a mechanism to compensate for the lack of anchoring fibrils. In contrast, skin sections from bullous pemphigoid patients with inflammatory subepidermal blistering did not show an upregulation of AMACO expression (not shown). However, the blister plane in bullous pemphigoid is expected to be on the side of the basement membrane facing the epithelium, not facing the dermis, where anchoring cords are localized. Interestingly, mutations in the AMACO gene were identified in a male with congenital anomalies of the kidneys and urinary tract (CAKUT) [30]. Therefore, defects in the formation of anchoring cords by AMACO and the Fraser complex proteins could be implicated in the pathogenesis of this disease. The importance of AMACO’s function could differ between tissues. It should be noted, as a limitation of the study, that we have not performed all forms of analysis on all tissue types and at all stages, and can therefore not exclude that there may be tissue and stage specific differences in the importance of AMACO’s function. In the future, an in depth immunogold electron microscopy analysis of anchoring cord assembly in, e.g., the kidneys and urinary tract, would be of interest.

Dermal–epidermal adhesion, and also the anchorage of other epithelia to the underlying tissue, is important both in development and in the adult organism. The present study builds on our recent demonstration of a novel anchoring structure, the anchoring cords, and shows that, at least at the dermal–epidermal junction, this structure can be formed without the peripheral component AMACO. This narrows down the group of proteins implicated in causing blistering skin diseases and thereby facilitates the molecular diagnosis of human patients.

## 4. Materials and Methods

### 4.1. Generation of an AMACO-Knockout Mouse Line

To generate AMACO-knockout mice, AMACO-KO ES cells (Vwa2tm1a(KOMP)Wtsi, RRID:MMRRC_064682-UCD), in which exon 3 had been successfully targeted, were ordered from the KOMP repository. Blastocyst injections into C57BL6/J foster mothers were performed at the Transgenic Core Facility of the MPI of Molecular Cell Biology and Genetics, in Dresden, Germany. Chimeric mice were crossed at the transgenic core facility of the Center for Molecular Medicine Cologne, Germany to generate germline mice. Exon 3 of the AMACO (VWA2) gene was deleted by crossing with a Cre deleter strain, to obtain a global knockout.

### 4.2. Animal Husbandry

Mice were bred to the C57BL/6N background and housed in a specific pathogen-free facility. The study protocol and all animal procedures were in compliance with the principles of laboratory animal care and the German laws on the protection of animals (§4 Abs.2 TierSchG). Animal protocols were approved by the veterinary agency of North-Rhine Westphalia (LANUV NRW, Recklinghausen, Germany). Mice were housed in individually ventilated cages and subjected to a 12:12 h light/dark cycle, at a temperature of 22 °C. Water and standard food were available ad libitum.

### 4.3. Genotyping of Mice

For genotyping of adult mice, ear biopsies were incubated with 300 μL lysis buffer (10 mM Tris, 100 mM EDTA, 0.5% SDS, pH 8.0) and 10 μL proteinase K (Qiagen, Hilden, Germany), at 55 °C overnight. The DNA was then precipitated with isopropanol, centrifuged for 10 min at 12,000× *g* at 4 °C, and the pellet washed with 70% ethanol. The DNA pellet was air dried and then dissolved in 50 μL of 5 mM Tris at pH 8.5. For this purpose, the samples were placed in a heated shaking incubator for at least 2 h. One microliter of the DNA sample was used for the genotyping PCR, with a primer pair designed for a specific region on exon 3 and the neo cassette, 5′-CACACCTCCCCCTGAACCTGAAAC-3′ (forward) and 5′-ATCCACCTCAACAGCAGACCAC-3′ (reverse). The primer pair 5′-GCTCCAAAAGCAGAGAGACAC-3′ (forward) and 5′-ATCCACCTCAACAGCAGACCAC-3′ (reverse) was used for the wildtype.

### 4.4. Isolation of Total RNA

Total RNA from newborn mice was isolated using TRIZOL (Invitrogen, Dreieich, Germany). A mass of 50 to 100 mg of each sample was homogenized in 1.5 mL of TRIZOL reagent. Chloroform (0.2 mL) was mixed with each 1 mL of TRIZOL reagent. The tubes were vortexed for 15 s, incubated at RT for 3 min, and centrifuged at 12,000× *g* for 15 min at 8 °C. The upper aqueous solution, containing RNA, was transferred into a fresh tube, and the RNA precipitated by dilution (1:2) with isopropanol. The samples were incubated for 10 min at 15 to 30 °C and centrifuged for 10 min at 12,000× *g* at 4 °C. The RNA pellet was washed repeatedly with 75% ethanol, re-dissolved in water, and RNA quantified by use of a spectrophotometer.

### 4.5. RT-PCR of Isolated mRNA

RNA was reverse transcribed to cDNA. RNA (1 μg) was mixed with 0.4 μL oligo (dT) primers (69 μM), 10 μL ddH2O, and incubated at 70 °C for 10 min, followed by 2 min on ice. After addition of 2 μL 5X “First Strand” buffer (Invitrogen, Dreieich, Germany), 1 μL DTT (100 mM), 0.4 μL dNTPMix (25 mM), 0.2 μL RNase inhibitor, and 0.2 μL Superscript II, the synthesis reaction was carried out at 42 °C for 1 h, and inactivated at 70 °C for 15 min. Remaining RNA residues were removed by adding 1 μL RNase H, at 37 °C for 20 min. Finally, the cDNA was diluted 1:3 with ddH2O. PCR was performed using the primer pairs 5′-CTAACAACATGCCTCCACTTC-3′ (forward) and 5′- TGAGAGCCATCTAACAGAAACAGG-3′ (reverse), for exon 1 to exon 3, 5′-TTGCCAGTGAGCGAGCGAG-3′ (forward) and 5′-TGCTTTCCTTCACTTCCTGTC-3′ (reverse) for 5′UTR to exon 4, 5′-CGACAGGAAGTGAAGGAAAG-3′ (forward) and 5′-AGCGTGATTCAGTGAGCAG-3′ (reverse) for exon 4 to exon 10 and 5′-AGAGGATCAAGGCAAGCAG-3′ (forward) and 5′-ACCGTCATCACCTTGTCCTC-3′ (reverse) for exon 7 to exon 11.

### 4.6. SDS-Polyacrylamide Gel Electrophoresis and Immunoblotting

Tissue was homogenized and extracted in 150 mM NaCl, 2 mM EDTA, 1% Nonidet P-40, 50 mM Tris/HCl pH 7.4, and complete protease inhibitor (Roche Diagnostics, Mannheim, Germany). Extracts were subjected to SDS-polyacrylamide gel electrophoresis on 4–12% polyacrylamide gradient gels. Resolved proteins were electrophoretically transferred to a nitrocellulose membrane and loading controlled by Ponceau S staining. The nitrocellulose membrane was incubated in 5% (*w*/*v*) milk powder in TBS, for 1 h at RT. Primary antibodies (Table 1) were prepared by dilution in 5% milk powder (*w*/*v*) in TBS, and incubated with the membrane for either 1 h at RT or overnight at 4 °C. The membrane was washed three times with TBS with 0.1% (*v*/*v*) Tween-20 (TBS-T). Secondary antibodies were diluted again in 5% milk powder and incubated with membrane, for 1 h at RT. The membrane was again repeatedly washed, incubated with ECL solution for 5 min at RT, exposed on x-ray film, and developed.

### 4.7. Immunofluorescence Microscopy

Cryosections were washed with PBS to remove the Tissue Tek, fixed with 2% paraformaldehyde in PBS for 10 min, and washed three times in PBS. For dewaxing, paraffin-embedded sections were incubated in xylol followed by a descending alcohol series, rinsed with water, incubated with hyaluronidase buffer for 30 min at 37 °C, for antigen retrieval, washed twice with TBS for 5 min each, and blocked with 5% BSA in 0.1% Triton in TBS at RT for 1 h. The primary antibodies (Table 1) were applied for 1 h at RT, the sections washed, secondary antibody applied, and the sections again washed. Pictures were taken with a confocal laser scanning microscope (Leica, Wetzlar Germany).

### 4.8. Hematoxylin and Eosin Staining

Paraffin-embedded sections were deparaffinised, immersed in a hematoxylin solution for 3 min, rinsed briefly in tap water, quickly immersed in HCl–alcohol, rinsed in water, and incubated in eosin solution for 3 min. The sections were again rinsed in water, immersed in 70% ethanol, followed by 80% and 96% ethanol for 3 min each, rinsed twice in either isopropanol or 100% ethanol, twice in xylol, and finally covered with DPX (dibutylphthalate polystyrene xylene).

### 4.9. Electron Microscopy

Wildtype and AMACO-deficient newborn mouse back skin was excised and carefully sliced into 1 mm cubes and stored in either Dulbecco’s Modified Eagle Medium (DMEM) or Michel’s Buffer. The skin samples were subsequently incubated in a solution of a polyclonal affinity-purified antibody (Table 1), diluted 1:5 in serum-free DMEM, overnight at 4 °C. The tissue cubes were washed in DMEM for 4 h and subsequently immersed in a 1 nm gold-labeled diluted secondary antibody suspension (Aurion GAR ultrasmall, Electron Microscopy Sciences, Hatfield, Pa), overnight at 4 °C. After a rigorous wash in DMEM, the tissues were immersed in gold enhancement solution (Nanoprobes, Yaphank, NY), rinsed, fixed in 1.5% glutaraldehyde, 1.5% paraformaldehyde with 0.05% tannic acid, post-fixed in 1% osmium tetroxide, and then dehydrated and embedded in Spurr’s epoxy. The stained sections were examined using either a Philips EM410LS or a FEI Tecnai G2 transmission electron microscope (Thermofisher, Hillsboro, Or).

## Figures and Tables

**Table 1:** Primary antibodies.

Antibody ^1^	Origin Species	Host Species	Source
AMACO	Mouse	Guinea Pig	[7]
Frem1	Mouse	Rabbit	[21]
Frem2	Mouse	Rabbit	[21]
Fras1	Mouse	Rabbit	[4]
Npnt	Mouse	Rabbit	[30]

^1^ All antibodies were affinity purified.

## Data Availability

The data presented in this study are available in the article.
